# Attentional mechanisms in light training tasks

**DOI:** 10.3389/fspor.2025.1623558

**Published:** 2025-08-22

**Authors:** Andria Shimi, Theofanis Kyriacou, Marios N. Avraamides

**Affiliations:** ^1^ Department of Psychology, University of Cyprus, Nicosia, Cyprus; ^2^ CYENS Centre of Excellence, Nicosia, Cyprus; ^3^ Department of Experimental Psychology, University of Oxford, Oxford, United Kingdom

**Keywords:** attention, attentional orienting, visual search, motion object tracking, cognition, reactive agility, sports, light training

## Abstract

**Introduction:**

In this study, we investigated the involvement of different aspects of attention in a light training task requiring fast physical responses to targets.

**Methods:**

Fifty adult participants carried out drills in SpeedPad, a Virtual Reality (VR) adaptation of the Batak Pro and the Fitlight Trainer systems commonly used by athletes of various sports. Participants also carried out three established cognitive tasks on a desktop computer: the Posner cueing task, a visual conjunction search task, and a Motion Object Tracking (MOT) task.

**Results:**

Results revealed significant correlations among performance on all four tasks, aligning with theoretical expectations. Regression analyses conducted for four array sizes in SpeedPad with 9, 15, 19, and 24 possible target locations, showed that the speed of attentional orienting to a target, measured with the Posner cueing task, was a significant predictor of SpeedPad performance across all array sizes. Accuracy in the MOT, which required splitting attention across multiple target locations and tracking moving targets, significantly predicted SpeedPad performance for array sizes 15, 19, and 24. However, the speed of visual search did not account for additional variance in SpeedPad performance beyond that explained by the other variables.

**Discussion:**

Overall, our results indicate that light training tasks like the SpeedPad rely on the efficient deployment of cognitive processes such as the spatial orienting of attention and the ability to split attention across multiple locations in the environment. These findings highlight the importance of cognitive skills for reacting fast to objects, suggesting that VR light training tasks could serve as valuable tools for exercising both cognitive and physiological processes in athletes across various sports.

## Attentional mechanisms in light training tasks

Although athletes and trainers have traditionally emphasised the development of physical skills and technique to enhance athletic performance, recent years have seen a growing focus on improving mental skills as well. This shift is unsurprising given that mental skills are fundamental to many tasks athletes perform during competition and training.

Consider, for example, the case of a soccer midfielder whose task is to pass the ball to a teammate further up the field. At first glance, this may seem like a simple task relying solely on physical skills and technique. However, a closer examination reveals its complexity. To pass the ball successfully, the midfielder must first determine the positions of both teammates and opponents, which requires visually tracking the movements of multiple players simultaneously and, in some cases, estimating their trajectories using information from memory. Before deciding where to pass the ball, the player must also visually scan the field to locate an unmarked teammate, a process that depends on the ability to shift attention efficiently from one location to another. This example illustrates that even seemingly simple decisions in soccer, such as passing the ball to a teammate, rely heavily on cognitive abilities. Thus, efficient use of attention, perception, and memory is crucial for processing information and supporting rapid decision-making on the football pitch (1, 2).

Indeed, past research has documented the important role of cognitive skills in sports; see Millard et al. (3) for a review of skills relating to vision and Mann et al. (2) for a meta-analysis of perceptual-cognitive factors in sport. Moreover, trainers and athletes frequently incorporate training exercises that appear to depend, at least partly, on cognitive processes. A notable example is the widely used reactive agility paradigm.

In a typical reactive agility drill, the athlete initially sprints forward but then changes direction in response to a stimulus, e.g., a flashing light indicating left or right or a signal from the trainer (4). Beyond physical agility, performance in such drills depends on the ability to make fast decisions about direction changes, which are influenced by cognitive and perceptual factors (4, 5). These include the detection and perceptual processing of the stimulus, the bottom-up or top-down orienting of attention (depending on the details of the task), and the execution of motor responses.

Past research in sports science has shown that performance in reactive agility drills can capture differences across athletes at different levels. For example, Gabbett and Benton (5) demonstrated that performance on a reactive agility test, requiring athletes to change sprint direction in response to the direction of movement by the investigator, distinguished higher-skilled rugby players from less-skilled ones. Similarly, Lockie et al. (6), found that a reactive agility test could differentiate semiprofessional basketball players from amateurs. Notably, performance on a planned version of the same test, in which participants knew the direction change in advance, did not predict the skill level of the player.

Overall, past findings suggest that reactive agility, as measured by drills requiring changes in sprint direction in response to a stimulus, is a critical ability to cultivate for several sports. Indeed, in sports like rugby, basketball, and football, athletes frequently need to change movement direction unexpectedly during the game, performing a task closely resembling those practiced in reactive agility drills. If these drills primarily train cognitive processes, such as the ability to detect and respond as fast as possible to abrupt stimuli (in addition to motor skills), then similar benefits may also be achieved with training tools that involve rapid responses to sudden stimuli, even without significant whole-body movement.

One popular tool fitting this description is the Batak machine (https://www.batak.com/). In its standard configuration, the Batak features an array of 8 or 12 lights arranged vertically, in front of the athlete, in a fixed configuration that allows for maximum stretching. Target lights light up sequentially, requiring the athlete to touch them as quickly as possible. Like reactive agility drills, a Batak session involves fast responses to stimuli, although the whole-body movement it entails is rather minimal^1^. A more modern implementation of the Batak concept is the Fitlight Trainer system (FITLIGHT Sports Corp. Canada), which uses individual wireless LED lights that can be placed anywhere around the athlete. Thus, depending on the setup, the Fitlight system can involve more extensive movement.

Research with lighting training tools such as the Batak and the FITLIGHT systems has documented that, like whole-body reactive agility tasks, these tools possess discriminatory power. For example, Zwierko et al. (7) found that handball players demonstrated significantly faster reaction times than non-athlete controls when carrying out exercises with Fitlight lights that were arranged horizontally in front of them. Similarly, in a study with 119 students aged 10–12, Reigal et al. (8) reported that the extent of engagement in physical activity predicted reaction times in both a simple version of Fitlight, involving responses to target lights with the dominant hand, and a complex version requiring responses with different hands based on the color of the lit light. Notably, only reaction times in the complex task correlated with scores on the D2 test, a paper-and-pencil neuropsychological measure of selective and sustained attention.

Past research has also highlighted benefits with light training across various measures. For example, Arede and Carvalho (9) tested 22 athletes from diverse sports (soccer, table tennis, basketball, athletics, and handball) and found that those who completed biweekly training with the Batak Pro for 12 weeks, in addition to their sport-specific training, exhibited significant improvements in motor skills, and particularly in dynamic stability, compared to an active control group that performed only sport-specific training. However, no improvements were found for either group in reactive agility drills requiring whole-body movement. Similarly, Hassan et al. (10) showed that basketball players who trained in an 8-week program that included Fitlight exercises exhibited greater improvements in dribbling skill (assessed by a modified agility *t*-test) and hand reaction time (assessed with a visual reaction time apparatus) than players who completed the same training program without Fitlight exercises.

While several studies suggest that light training may improve athletic performance, others report different results. For example, Theofilou et al. (11) found no benefit of training with Fitlight. In this study, 38 soccer players aged 10–15 were divided into two groups. The control group followed their regular soccer training program for 6 months, while the intervention group carried out the same program with the addition of Fitlight training 5 times per week for 15 min per session. Cognitive function and physical fitness were assessed before and after the 6-month period. Although both groups showed within-group improvements in several measurements (e.g., reaction speed in a Pen-to-Point test and accuracy in a Figure Drawing test), no significant differences were found between the two groups post-intervention.

We conjecture that the mixed results in the literature are due to the use of different tests to assess performance in past studies. More importantly, the selection of dependent measures in many studies appears to overlook the specific cognitive processes targeted by the intervention. For example, Theofilou et al. (11) assessed cognitive function with the Figure Drawing test and the Pen-to-Point-test, both taken from the Cognitive Function Scanner Mobile Test Suite. In the Figure Drawing test, participants trace a curved line presented on a tablet using a digital pen, while in the Pen-to-Point test, they use the pen to point at the centre of small crosses arranged in a straight line on the screen. Although these tasks assess aspects of eye-hand coordination, this ability does not seem directly relevant to light training, potentially explaining the lack of group differences in the study. Moreover, even in studies demonstrating benefits of light training, it remains unclear whether these improvements stem primarily from enhancements in physical skill and agility or from cognitive factors. This ambiguity arises because little research has specifically examined the cognitive mechanisms underpinning the effectiveness of these training tools.

Here, we argue that before evaluating the potential benefits of light training, it is essential to take a step back. We posit that we must first gain a more nuanced understanding of the cognitive and physical processes that these tools tap on, before we go on to assess how they can improve athletic performance. By identifying the underlying mechanisms, we can then design training studies that specifically assess improvements in activities that rely on the same processes that light training implicates. This approach will also provide valuable guidance for athletes and coaches, helping to determine which sports and which specific activities can benefit from light training.

As a first step in this approach, the present study examined the cognitive skills underlying performance in a light training tool, focusing on attention. We utilised the SpeedPad app (MentisVR Ltd), a modern and flexible implementation of the light training paradigm that employs immersive Virtual Reality (VR) technology to position users in front of virtual light configurations. VR technology is becoming increasingly popular in sports training as it allows athletes to train in realistic settings from anywhere [see (12), for a systematic review of VR use in sports education and training and Craig (1), for a discussion on the benefits of VR for cognitive training in sports].

SpeedPad enables extending the number of light targets beyond those available in Batak and Fitlight, allowing the user to carry out drills with and without whole-body movement, as well as drills that rely on peripheral vision (i.e., by placing targets outside the central field of view). Previous research from our group with non-athletes (13), has shown that scores from an 1-minute SpeedPad drill predicted performance in a goalkeeping task, suggesting that the task implicates cognitive processes that are important to goalkeeping, e.g., the orienting of attention [see (14), for evidence about the cognitive processes underlying goalkeeping].

Based on our past results with SpeedPad, we focus here on three aspects of attention that we hypothesize may drive performance in this app: the abilities to split attention across multiple locations, to search a visual array for a target, and to orient attention to a target. Using regression analyses, we examined the contributions of these cognitive processes to reaction time (RT) for various array set sizes in light training with SpeedPad. As we considered the ability to orient attention the most relevant to SpeedPad, we added this measure in the first step of the regression analysis. In a second step, we added together two additional measures, one for each of the abilities to visually search for a target and to split attention to multiple targets. This allowed us to evaluate whether these two processes would explain variation in SpeedPad performance over and above the variation accounted for by attentional orienting.

Given that our aim was to examine the cognitive processes underlying the execution of this task, we did not impose any restrictions for the recruitment of participants. Yet, for the purpose of the statistical analyses, we took into consideration whether participants engaged in sports or other physical activities such as dancing. In particular, we examined whether participants reporting engagement in physical activity would outperform those who did not, in SpeedPad performance.

## Method

### Participants

Fifty healthy adult participants (25 male and 25 female; mean age = 23.12 years, SD = 4.38) participated in the study. A power analysis using G*Power revealed that 42 participants were needed to detect a medium-to-large effect size f^2^ = 0.25 in a stepwise regression analysis with 1 predictor included in the first step and 2 additional predictors in the second step, assuming *α* = 0.05 and power = 0.80. Participants were recruited through social media advertisements and word of mouth. Although no selection criteria in terms of sport participation and engagement in physical activity were enforced, 16 participants reported that they engaged, currently or within the last 2 years, in various athletic or dancing activities. Specifically, in these 16 participants, there were 2 current and 2 former soccer players, 1 current and 1 former track and field athletes, 1 current taekwondo athlete, 1 former judo athlete, 1 current Greco-roman wrestler, 1 current water polo athlete, 1 current handball athlete, 1 current cross-fit athlete, 3 former ballet dancers, and 1 former break dancer. All current and former athletes were or had been at the collegiate level, and none of them competed professionally. All participants had normal or corrected-to-normal vision and reported no colour vision deficits. They all signed a consent form prior to participation and were thoroughly debriefed afterwards. Participants received €10 for their participation. The study was ethically approved by the Cyprus National Bioethics Committee prior to initiation.

### Materials

**Figure 1:**
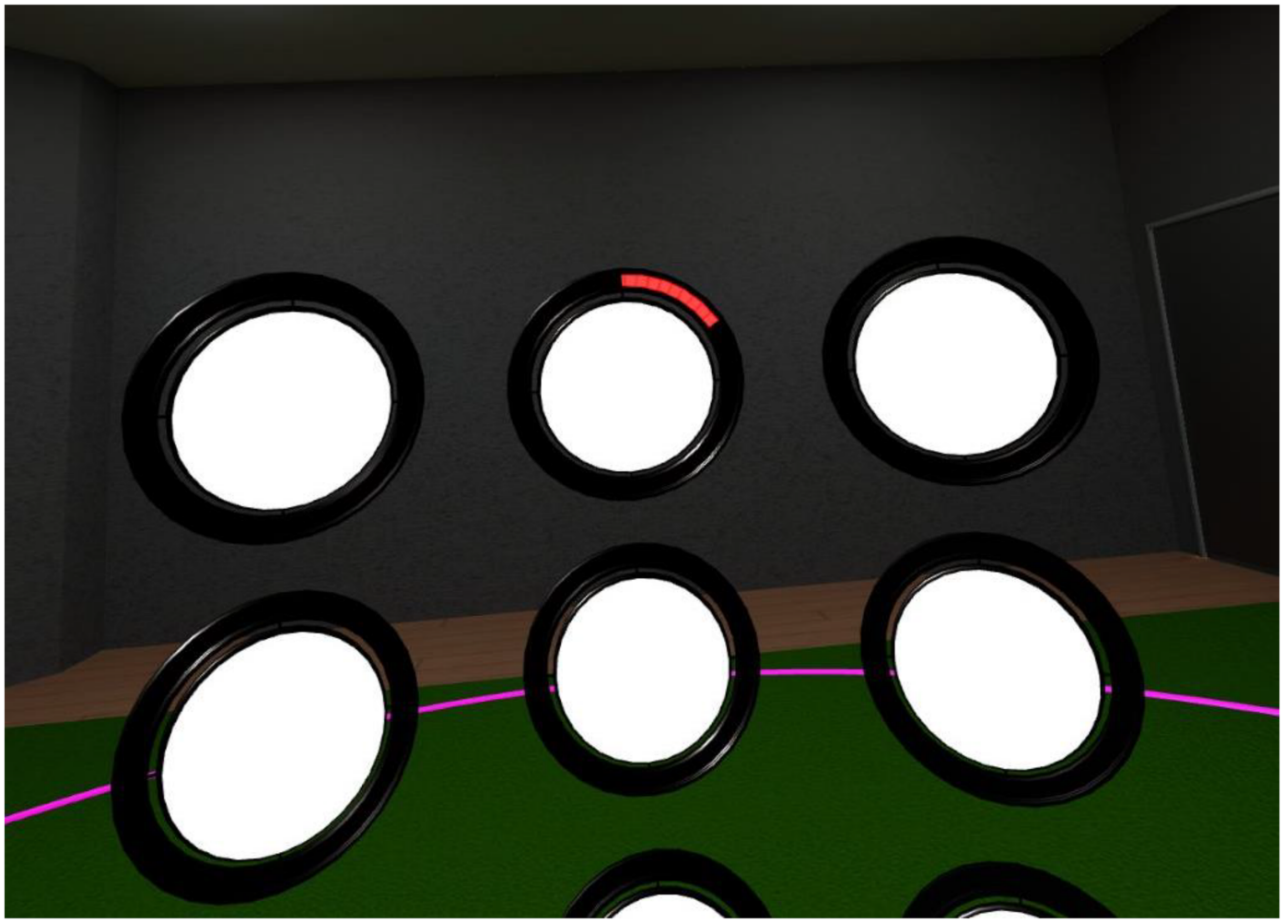
Screenshot from SpeedPad game play. Users are presented with an array of discs. When the outline of the disc starts becoming red, participants can hit the target with the handheld controller.

SpeedPad. This is a Mixed Reality task developed by MentisVR Ltd (http://mentis-vr.com). It is an adaptation of the Batak Pro machine (Quatronics Ltd.) and the Fitlight Trainer system (Fitlighttraining.com), both widely used for reaction-speed training in various sports. In SpeedPad, participants are presented with an array of white circular discs with a black outline and move their arms to hit, as fast as possible, each disc whose outline changes gradually to red (Figure 1). The target remains available for 7 s, which is how much time its outline takes to fill completely with red. Participants can respond any time within these 7 s. As soon as they respond, the target disc reverts to white, and another disc starts turning red. Performance is measured by counting the number of target discs correctly hit within a set time. SpeedPad operates in two modes, either in Mixed Reality where the virtual discs are integrated into the real-world environment or in Virtual Reality (VR) where participants interact in a fully virtual environment. In the current study, we used the VR mode and presented the discs in a training gym environment.

Posner cueing task. This is a widely used task aimed to measure the speed at which individuals orient their covert attention (i.e., attention without moving the eyes) to stimuli (15). In this task, participants respond to a target that appears on the computer screen either to the left or right of a central fixation cross. In our implementation, the target was a red circle that flashed on the screen. Before the target appeared, an arrow was briefly presented at the centre of the screen, pointing either to the left or right. In valid cue trials, the target appeared on the side cued by the arrow, while in invalid cue trials, it appeared on the opposite side. Typically, there are more valid than invalid trials, often with a 4:1 ratio, to encourage participants to trust the cue and shift attention in a top-down manner. A common analysis involves subtracting the reaction time (RT) for valid cue trials from the RT of invalid trials to calculate a cue benefit score, which reflects the facilitation of stimulus processing by the cue. The version of the task we used was developed in PsychoPy and was a modified version of a task retrieved from https://gitlab.pavlovia.org/P1NKBOW/posner.

Visual Search Task. This task measures the ability to locate a target amongst distractors. In the version we used, participants viewed displays where the target, i.e., the letter T, was presented in one of four possible orientations (upright, upside down, or rotated 90° from the upright to the left or right). The target was embedded amongst distractors, i.e., the letter L, with the number of distractors varying across trials. Displays could include 2, 6, 10, 14, or 18 distractors in addition to the target. In these spatial configuration trials (where the target is defined by a spatial combination of features that are also present in the distractors, i.e., a horizontal and a vertical line segment), RT typically increases with the size of the display (i.e., the number of the distractors), a result known as the *display size* effect. The slope of this effect indicates how the number of distractors impacts the efficiency of visual search. The version of the task we used was developed in PsychoPy by Colin Quirk and was retrieved from https://github.com/colinquirk/templateexperiments.

Multiple Object Tracking. This task measures the ability to simultaneously track the trajectories of multiple moving targets. In the version we used, participants first viewed a fixation cross for 2 s followed by 12 static orange circles. Subsequently, 2, 3 or 4 of these circles flashed green for 1 s before returning to orange. Then, all the circles began moving across the screen, and participants were instructed to track the previously green circles. After 10 s, the circles stopped moving, and one was highlighted in red. Participants indicated whether the red circle was one of the targets by pressing *p* for “yes” and q for “no” on the keyboard. Response accuracy and RT was recorded. The task was developed in OpenSesame by Wardhani et al. (16).

### Procedure

Participants were tested individually at a quiet laboratory at the University of Cyprus and sat at a comfortable distance from the computer screen in the 3 computerised tasks. Participants completed the 4 tasks in the same order, i.e., first the Posner cueing task, then the Visual search task followed by the MOT, and finally the SpeedPad. Self-paced breaks were inserted after each task. Before carrying out the computerized tasks, they were asked to provide information about whether they engaged in a sport, and if yes, to provide details on their engagement, i.e., which sport, for how long, and at what level.

First, participants carried out the Posner cueing task. They were instructed to rest their index finger on the “k” key on the keyboard and their middle finger on the “j” key. Each trial began with a fixation cross presented for 800 ms, followed by a centrally presented blue arrow (cue) for 400 ms, pointing either to the left or right. Next, a red circle (target) was presented for 200 ms on either the left or right side of the fixation cross. Participants responded to the target as fast as possible by pressing the “k” key if the circle appeared on the left or the “j” key if the circle appeared on the right. The task consisted of 120 trials, with 96 valid trials (80%) and 24 invalid trials (20%). Before the experimental trials, participants completed 10 practice trials to familiarize themselves with the task. Participants received feedback about the accuracy of their responses in the practice trials but not in the experimental trials.

After completing the Posner cueing task, participants took a short self-paced break before proceeding to the Visual Search task. In each trial of the Visual Search task, they searched for the target—the letter T in one of four orientations—among distractors, which were 2, 6, 10, 14, or 18 instances of the letter L. Participants responded by pressing the arrow key on the keyboard corresponding to the target's orientation. For example, if the T was rotated 90° to the left, they pressed the left arrow key. The search display remained visible until participants responded, and they were instructed to respond as fast as possible. The task consisted of 120 trials, divided into six blocks, with 24 trials for each display size condition. Trials from the 5 display size conditions were randomized across the task. Prior to the experimental trials, participants completed 5 practice trials, one for each display size condition, and received feedback for the accuracy of their response.

Next, participants carried out the MOT task. Here, they tracked the trajectory of 2, 3, or 4 circles, out of 10 moving circles for 10s. When the movement stopped, one circle was highlighted, and participants were pressed the “p” key to indicate if it was a target or the “q” key to indicate that it was not. The task consisted of 30 trials, with 10 trials for each target load. Prior to experimental trials, participants completed 3 practice trials to familiarize themselves with the task. As in the other tasks, participants were instructed to respond as fast as possible. Participants received feedback on the accuracy and reaction time for each trial, in both the practice and experimental sessions.

Finally, participants carried out four 45 s sessions of SpeedPad. In each session, participants moved their arms to hit discs that changed colour as fast as possible. The first session involved 9 discs (i.e., set size 9), the second 15 (i.e., set size 15), the third 19 (i.e., set size 19), and the last 24 discs (i.e., set size 24). Before the experimental sessions, participants completed a 30 s practice session with 9 discs. Their performance was measured as the number of target discs successfully hit within the 45 s session. Participants received feedback about their score after each session.

### Statistical analyses

All analyses were carried out using the jamovi software package (17). We first examined whether participants' reported current and prior involvement in physical activities influenced their performance on the SpeedPad task. To this purpose, we classified participants into two groups: those who reported current or prior engagement in dance and sports (16 participants) vs. those who did not (34 participants). We then carried out a Repeated-Measures Analysis of Variance (ANOVA) on SpeedPad scores with terms for engagement in physical exercise and SpeedPad size. This analysis allowed us to examine whether performance on SpeedPad differed across the two groups.

We then analyzed performance on each of the computerized tasks to validate that they would yield the expected pattern of results, based on the literature. This also allowed us to confirm that the fixed order in which the tasks were administered did not produce carry-over effects that would change the typical pattern of results obtained with these tasks. For the Posner Cueing task, we carried out a paired-wise t-test to compare reaction times (RTs) for valid and invalid cue trials, confirming the presence of a cueing effect. For the Visual Search task, we carried out a Repeated-Measures ANOVA on RT with display size (2, 6, 10, 14, or 18 distractors) as the independent variable. This allowed us to verify that the expected display size effect (i.e., RT increasing with the number of distractors) was present in our data. For the MOT task, we ran a repeated-measures ANOVAs for accuracy and RT with set size (2, 3, or 4 targets). This analysis allowed to assess whether performance at 4 targets, which is the capacity limit for most people, would be inferior to performance for 2 and 3 targets.

For all ANOVA tests conducted, we tested for homogeneity of variances using Levene's test. Sphericity violations were assessed using Mauchly's W and were corrected, whenever needed, by applying a Greenhouse-Geisser correction. *post-hoc* comparisons were made with Tukey corrections.

Subsequently, we conducted correlational analyses to examine the relation between SpeedPad scores and measures from the 3 computerized tasks. Finally, we carried out stepwise regression analyses—separate for each SpeedPad set size—to examine how much of the variance in SpeedPad would be accounted for initially by performance in the Cueing task (Step 1) and then by performance in the Visual Search and the MOT (Step 2). We used the Autocorrelation test to verify that the residuals from the regression models were independent from each other, and we also tested for normality and collinearity. All assumptions for each model, one for each of the 4 SpedPad set sizes, were met.

## Results

### Engagement in physical exercise

**Figure 2:**
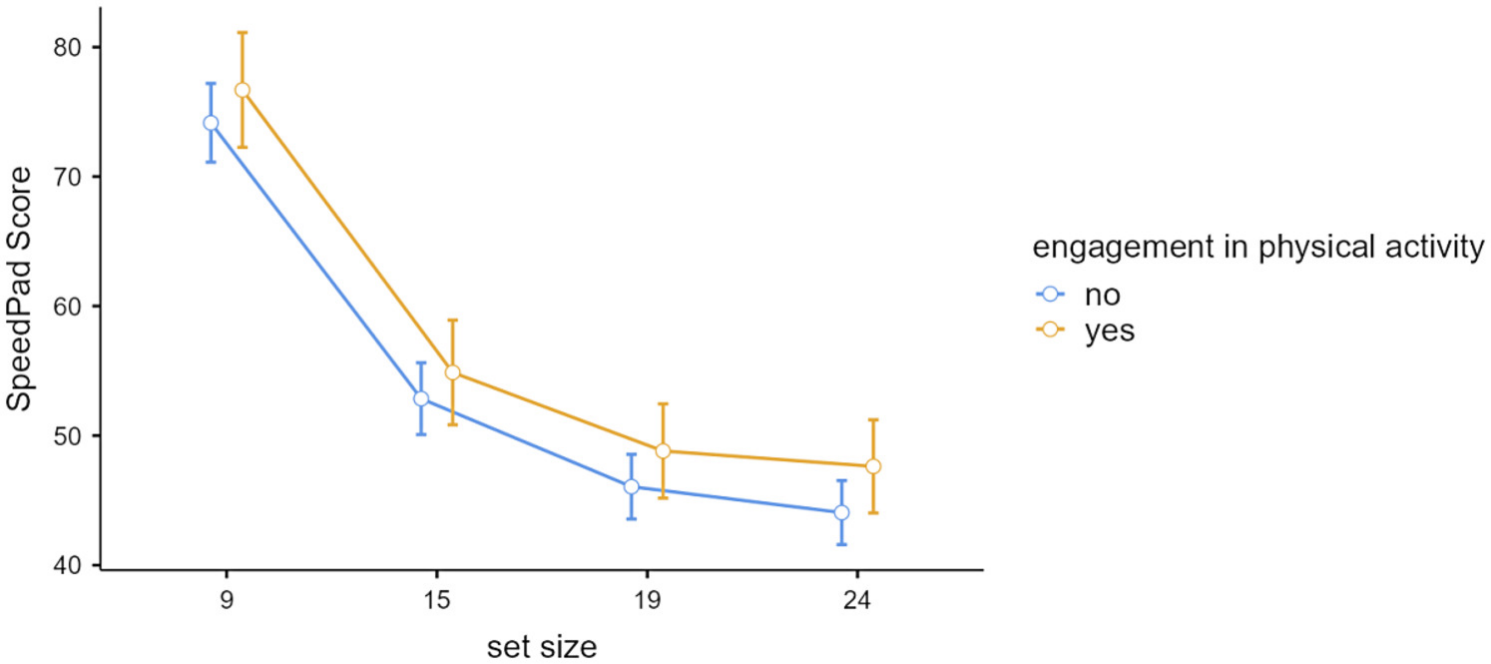
Speedpad score as a function of set size and engagement in physical activity. Error bars represent 95% Confidence Intervals.

A repeated-measures ANOVA with factors for sports/dance-engagement (yes vs. no) and SpeedPad set size (9, 15, 19, 24) revealed that although participants who engaged in physical activity hit numerically more discs (M = 57) than those who did not (M = 54.28), the difference was not statistically significant, *F*(1,48) = 1.6, *p* = .21, *η²* = .009. There was also no interaction between physical activity engagement and SpeedPad set size, *F*(3,144) = 0.30, *p* = .82, *η²* = 0. However, the main effect of set size was significant, *F*(3,144) = 548.85, *p* < .001, *η²* = 0.667. As shown in Figure 2, SpeedPad scores decreased significantly as the number of discs in the set increased, consistent with expectations.

### Posner cueing task

A paired-sample *t*-test on RT for correct responses indicated a significant difference between trials with valid vs. invalid cues, *t*(49) = 9.82, *p* < .001, *d* = 1.39. As expected, participants responded faster in trials with valid cues (M = 281 ms) than in trials with invalid cues (M = 326 ms). The presence of a cue benefit confirmed that the task functioned as intended.

As shown in Table 1, the cueing benefit did not correlate with scores in any SpeedPad size. However, significant negative correlations were observed between SpeedPad scores and the RT for both valid and invalid cue trials, as well as the overall mean RT for the task. All correlations were significant (*ps* < .05), except for a marginal correlation between set size 15 and RT for invalid trials (*p* = .06).

**Table 1:** Correlations of cueing RT scores and SpeedPad scores.

Measure	Statistic	Cue Benefit RT	Valid Cue RT	Invalid Cue RT	Mean Cueing RT	SpeedPad 9	SpeedPad 15	SpeedPad 19	SpeedPad 24
**Cue Benefit RT**	Pearson's r	—							
	*p*-value	—							
**Valid Cue RT**	Pearson's r	−0.304*	—						
	*p*-value	0.032	—						
**Invalid Cue RT**	Pearson's r	0.533***	0.644***	—					
	*p*-value	<.001	<.001	—					
**Mean Cueing RT**	Pearson's r	0.154	0.895***	0.918***	—				
	*p*-value	0.286	<.001	<.001	—				
**SpeedPad 9**	Pearson's r	0.019	−0.637***	−0.550***	−0.652***	—			
	*p*-value	0.894	<.001	<.001	<.001	—			
**SpeedPad 15**	Pearson's r	0.106	−0.395**	−0.265	−0.359*	0.716***	—		
	*p*-value	0.462	0.005	0.063	0.010	<.001	—		
**SpeedPad 19**	Pearson's r	0.136	−0.463***	−0.302*	−0.417**	0.739***	0.818***	—	
	*p*-value	0.346	<.001	0.033	0.003	<.001	<.001	—	
**SpeedPad 24**	Pearson's r	0.049	−0.541***	−0.441**	−0.538***	0.784***	0.755***	0.840***	—
	*p*-value	0.735	<.001	0.001	<.001	<.001	<.001	<.001	—

* *p* < .05.

** *p* < .01.

*** *p* < .001.

### Visual search

**Figure 3:**
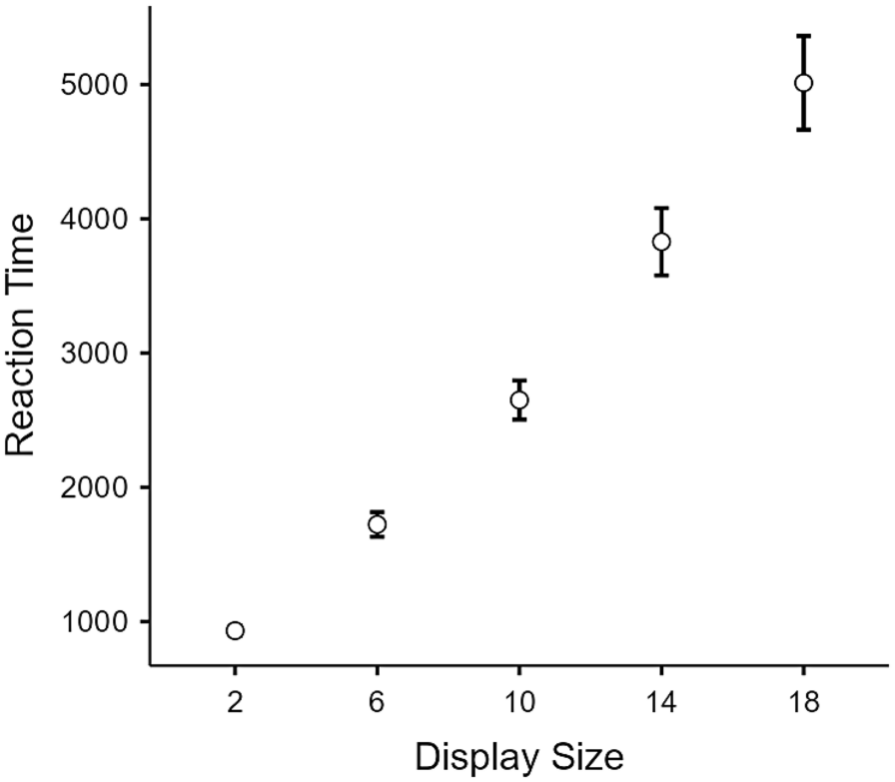
Reaction time in the visual search task as a function of display size. Error bars represent 95% Confidence Intervals.

First, a repeated-measures ANOVA confirmed the expected display size effect as RT increased linearly with display size, *F*(4,49) = 376.62, *p* < .001, *η²* = 0.802 (Figure 3).

We then computed the RT x set size function, yielding an intercept value and a slope value for each participant. The intercept represents baseline processing speed, reflecting the time required to make a decision without the influence of distractors. In contrast, the slope indicates the increase in RT for each additional distractor, with smaller slope values reflecting greater search efficiency and less impact from additional distractors.

As shown in Table 2, intercepts and slopes were strongly negatively correlated, *r*(48) = −.77, *p* < .001, indicating that participants with slower baseline processing speeds were less efficient when distractors were added to the display. Importantly, baseline processing speed in visual search did not correlate with SpeedPad performance. However, search efficiency (as indexed by the slope of the visual search function) was significantly correlated with SpeedPad scores. As seen in Table 2, negative correlations were observed between search slopes and SpeedPad scores, with a smaller slope (indicating higher efficiency) associated with better SpeedPad performance. All correlations were significant (*ps* < .05), except for the correlation with SpeedPad set size 19, which fell short of the alpha value, *p* = .09.

**Table 2:** Correlations of Visual Search (VS) slope and intercept with SpeedPad scores.

Measure	Statistic	SpeedPad 9	SpeedPad 15	SpeedPad 19	SpeedPad 24	VS intercept	VS Slope
SpeedPad 9	Pearson's r	—					
	df	—					
	*p*-value	—					
SpeedPad 15	Pearson's r	0.716***	—				
	df	48	—				
	*p*-value	<.001	—				
SpeedPad 19	Pearson's r	0.739***	0.818***	—			
	df	48	48	—			
	*p*-value	<.001	<.001	—			
SpeedPad 24	Pearson's r	0.784***	0.755***	0.840***	—		
	df	48	48	48	—		
	*p*-value	<.001	<.001	<.001	—		
VS intercept	Pearson's r	0.122	0.090	0.068	0.095	—	
	df	48	48	48	48	—	
	*p*-value	0.399	0.536	0.637	0.512	—	
VS slope	Pearson's r	−0.336*	−0.280*	−0.243	−0.285*	−0.771***	—
	df	48	48	48	48	48	—
	*p*-value	0.017	0.049	0.090	0.045	<.001	—

* *p* < .05

** *p* < .01

*** *p* < .001

### Multiple object tracking

As expected, performance on the MOT task decreased as the number of targets to be tracked increased. Accuracy dropped from.85 with 2 targets to.76 with 3 targets, and further to.70 with 4 targets. A repeated-measures ANOVA revealed a significant effect of the number of targets, *F*(2,98) = .18.12, *p* < .001, *η²* = .172. *post-hoc* comparisons with Tukey corrections confirmed that all pairwise differences were significant. RT increased from 940 ms with 2 targets to 1,012 ms with 3 targets, and further to 1,204 ms with 4 targets. A repeated-measures ANOVA on RT corroborated this pattern, revealing a significant main effect of the target number, *F*(2,98) = 12.33, *p* < .001, *η²* = .066. However, *post-hoc* comparisons showed that while the increase in RT from 3–4 targets was significant (*p* = .01), the increase from 2–3 targets was not (*p* = .21).

As shown in Table 3, several significant correlations were observed between SpeedPad scores and both accuracy and RT scores in the MOT. Notably, SpeedPad scores were positively correlated with accuracy scores and negatively correlated with RT, indicating that better SpeedPad performance was associated with higher accuracy and faster responses in the MOT task.

**Table 3:** Correlations of MOT Accuracy (acc), MOT R, and SpeedPad scores. Numbers at MOT labels indicate number of targets.

Measure	Statistic	MOT acc 2	MOT acc 3	MOT acc 4	MOT RT 2	MOT RT 3	MOT RT 4	MOT acc	MOT RT
MOT acc 2	Pearson's r	—							
	*p*-value	—							
MOT acc 3	Pearson's r	0.039	—						
	*p*-value	0.790	—						
MOT acc 4	Pearson's r	0.312*	0.202	—					
	*p*-value	0.027	0.160	—					
MOT RT 2	Pearson's r	−0.288*	−0.259	−0.014	—				
	*p*-value	0.043	0.070	0.924	—				
MOT RT 3	Pearson's r	−0.331*	−0.298*	−0.143	0.673***	—			
	*p*-value	0.019	0.035	0.323	<.001	—			
MOT RT 4	Pearson's r	−0.209	−0.146	−0.006	0.675***	0.518***	—		
	*p*-value	0.144	0.311	0.968	<.001	<.001	—		
MOT Mean acc	Pearson's r	0.558***	0.718***	0.725***	−0.274	−0.377**	−0.172	—	
	*p*-value	<.001	<.001	<.001	0.054	0.007	0.233	—	
MOT Mean RT	Pearson's r	−0.310*	−0.258	−0.057	0.884***	0.818***	0.885***	−0.303*	—
	*p*-value	0.029	0.070	0.693	<.001	<.001	<.001	0.032	—
SpeedPad 9	Pearson's r	0.140	0.347*	0.094	−0.434**	−0.223	−0.273	0.310*	−0.350*
	*p*-value	0.332	0.013	0.516	0.002	0.119	0.055	0.029	0.013
SpeedPad 15	Pearson's r	0.191	0.385**	0.279	−0.292*	−0.189	−0.319*	0.442**	−0.315*
	*p*-value	0.184	0.006	0.050	0.039	0.190	0.024	0.001	0.026
SpeedPad 19	Pearson's r	0.080	0.446**	0.190	−0.357*	−0.205	−0.366**	0.393**	−0.364**
	*p*-value	0.582	0.001	0.186	0.011	0.154	0.009	0.005	0.009
SpeedPad24	Pearson's r	0.057	0.383**	0.194	−0.289*	−0.091	−0.245	0.348*	−0.243
	*p*-value	0.693	0.006	0.177	0.041	0.530	0.087	0.013	0.088

* *p* < .05.

** *p* < .01.

*** *p* < .001.

### Regression analyses

Overall, the correlational analyses revealed significant associations between SpeedPad performance and measures from the three computerized attention tasks. To further understand the cognitive processes recruited by SpeedPad, we conducted hierarchical regression analyses.

Separate analyses were performed for each SpeedPad set size, with predictors entered in two steps. In Step 1, we included the average RT from the Posner Cueing task^2^, as we considered the orienting of attention to targets to be the most relevant cognitive mechanism for the SpeedPad task. In Step 2, we simultaneously entered the accuracy of the MOT task and the average RT from the Visual Search task.

For set size 9, the regression analyses revealed that at Step 1, orienting contributed significantly to the regression model, [*F*(1, 48) = 35.4, *p* < .001], accounting for 42,5% of the variation in SpeedPad performance. Adding MOT and visual search slope in step 2 accounted for an additional 7,2% of the variance, resulting in a significant change in R^2^, *F*(2,46) = 3.29, *p* = .046. As shown in Table 4, when the three predictors were included in Step 2, only attentional orienting remained a significant predictor of SpeedPad performance.

**Table 4:** Hierarchical regression analysis on SpeedPad score set size 9.

Step 1 coefficients—SpeedPad 9				
**Predictor**	**Estimate**	**SE**	***t***	***p***
Intercept^a^	126	8.66	14.57	<.001
Cueing RT	−169	28.43	5.95	<.001
^a^Represents reference level				
Step 2 coefficients—SpeedPad 9				
**Predictor**	**Estimate**	**SE**	***t***	***p***
Intercept^a^	113.28	1,249	9.07	<.001
Cueing RT	−150.92	28.11	5.37	<.001
MOT Accuracy	17.10	9.88	1.73	.090
Visual Search Slope	−0.023	0.014	1.67	.10
^a^Represents reference level				

For set size 15, the initial model with attentional orienting entered in Step 1 was significant, [*F*(1,48) = 7.11, *p* = .01, accounting for only 13% of the variance. Adding MOT accuracy and visual search slope in Step 2 significantly improved the model fit, explaining an additional 17.6% of the variance, *F*(2,46) = 5.81, *p* < .006. As shown in Table 5, in this set size and in contrast to set size 9, MOT accuracy emerged as a significant predictor of SpeedPad performance alongside attentional orienting.

**Table 5:** Hierarchical regression analysis on SpeedPad score set size 15.

Step 1 coefficients—SpeedPad 15				
**Predictor**	**Estimate**	**SE**	***t***	***p***
Intercept^a^	79.2	9.69	8.17	<.001
Cueing RT	−84.9	31.82	2.67	.01
^a^Represents reference level				
Step 2 coefficients—SpeedPad 15				
**Predictor**	**Estimate**	**SE**	***t***	***p***
Intercept^a^	52.86	13.36	3.96	<.001
Cueing RT	−61.61	30.06	2.05	.046
MOT Accuracy	31.35	10.57	2.97	.005
Visual Search Slope	−0.019	0.015	1.31	.198
^a^Represents reference level				

Similar results were obtained for set size 19 (Table 6). In Step 1, the attentional orienting explained 17.4% of the variance in SpeedPad performance, *F*(1,48) = 10.08, *p* = .003. Adding MOT accuracy and visual search slope in Step 2 explained an additional 12.2% of the variance, yielding a significant change in R^2^, *F*(2,46) = 3.95, *p* = .03. In the final model, both attentional orienting and MOT accuracy were significant predictors of SpeedPad performance.

**Table 6:** Hierarchical regression analysis on SpeedPad score for array size 19.

Step 1 coefficients—SpeedPad 19				
**Predictor**	**Estimate**	**SE**	***t***	***p***
Intercept^a^	74	8.58	8.63	<.001
Cueing RT	−89.4	28.17	3.17	.003
^a^Represents reference level				
Step 2 coefficients—SpeedPad 19				
**Predictor**	**Estimate**	**SE**	***t***	***p***
Intercept^a^	53.30	12.23	4.36	<.001
Cueing RT	−72.38	27.51	−2.63	.012
MOT Accuracy	24.34	9.67	2.52	.015
Visual Search Slope	−0.013	0.013	−0.94	.353
^a^Represents reference level				

Finally, for set size 24 (Table 7), attentional orienting in Step 1 accounted for 28.9% of the variance, *F*(1,48) = 19.55, *p* < .001. Adding MOT accuracy and visual search slope in Step 2 introduced a significant change in R^2^, explaining an additional 8.8% of the variance, *F*(2,46) = 3.26, *p* = .047. In the final model, both attentional orienting and MOT accuracy significantly predicted SpeedPad performance.

**Table 7:** Hierarchical regression analysis on SpeedPad score set size 24.

Step 1 coefficients—SpeedPad 24				
**Predictor**	**Estimate**	**SE**	***t***	***p***
Intercept^a^	80.1	7.95	10.08	<.001
Cueing RT	−115.4	26.09	4.42	<.001
^a^Represents reference level				
Step 2 coefficients—SpeedPad 24				
**Predictor**	**Estimate**	**SE**	***t***	***p***
Intercept^a^	64.64	11.47	5.64	<.001
Cueing RT	−99.67	25.81	−3.86	<.001
MOT Accuracy	18.94	9.08	2.09	.042
Visual Search Slope	−0.015	0.013	−1.21	.188
^a^Represents reference level				

## Discussion

The goal of the present study was to determine which aspects of attention underlie the execution of LED light training tasks that require speeded reactive responses to stimuli. Our results demonstrate that reaction time for orienting attention to a stimulus (measured with the Posner Cueing task) and the accuracy of tracking multiple moving targets (measured with the MOT task) uniquely predicted performance in the SpeedPad task. In contrast, the ability to search for a target among distractors, despite correlating with SpeedPad reaction time, did not explain additional variance.

Interestingly, self-reported engagement in sports and dance did not influence SpeedPad performance, suggesting that its cognitive demands are not easily captured by physical skills alone. On one hand, this null result highlights that SpeedPad performance likely reflects specific attentional abilities rather than general athletic performance. On the other hand, as our primary aim in this study was not to assess the effects of athletic experience, we recruited participants from the general population. As a result, we had a small and rather diverse group of participants who engaged in sports and dancing, which precludes us from drawing safe conclusions about either the capacity of the SpeedPad task to discriminate athletes and non-athletes or its reliance on physical skills. Overall, though, our results suggest that light training tools like SpeedPad may target fundamental attentional abilities that are not necessarily tied to physical activity, making them potentially useful across various populations.

That attentional orienting and tracking moving targets explained unique variance in SpeedPad performance but visual search did not, aligns with previous findings suggesting that not all cognitive abilities generalize across tasks (18) and highlights the specificity of SpeedPad performance to attentional skills such as orienting and tracking, rather than general cognitive ability. This finding is consistent with studies using similar light training tools, such as Theofilou et al. (11), where cognitive performance assessed through unrelated tasks (e.g., tracing or pointing) failed to show significant improvements during training. Together, these results emphasize the importance of selecting dependent measures that align with the processes targeted by the intervention. While prior research has documented cognitive benefits from sports training (e.g., perceptual speed), our findings suggest that SpeedPad specifically targets attentional mechanisms, such as orienting and split attention, which may not generalize from general athletic experience (18).

Our findings indicate that light training tasks such as the SpeedPad, Fitlight, and Batak rely heavily on attentional orienting, the ability to direct attention quickly to an abrupt target. Given that attentional orienting is fundamental for success in many fast-paced sports, training through such tasks holds the potential to enhance athletic performance. This aligns with findings from reactive agility studies (5, 6), which demonstrated that athletes' ability to respond to abrupt and unpredictable cues distinguishes higher from lower skill levels. Similarly, our results reinforce the critical role of rapid stimulus detection and attentional shifts in real-world tasks like reactive agility, where physical and cognitive demands interact. The reliance on attentional orienting aligns with prior findings in sports, where the ability to rapidly direct attention to relevant cues has been linked to athletic expertise. For example, Abernethy (19) showed that expert but not novice squash players could pick up information from an opponent's early arm action to anticipate the direction and force of the stroke [see (20) for similar results with cricket players].

Importantly, our experiment revealed that attentional orienting to a target predicted SpeedPad performance, irrespective of cue validity. This result suggests that SpeedPad performance reflects the general efficiency of attentional orienting, rather than the ability to strategically leverage cues of predictive shifts. Notably, SpeedPad scores were significantly associated with reaction times for both valid and invalid trials in the Posner Cueing Task. This highlights that participants' ability to rapidly detect and shift attention, regardless of whether the cue correctly predicts the target, drives SpeedPad performance. This pattern suggests that participants who are generally faster at orienting their attention to a stimulus, whether the cue is valid or not, perform better on SpeedPad. The cueing benefit itself may not relate to SpeedPad because the task primarily demands rapid detection and response to salient targets, rather than leveraging predictive cues. In the SpeedPad task, participants anticipate that a disc will light up, which engages top-down attentional readiness, i.e., the goal-driven focus needed to prepare for the stimulus. However, the moment the disc changes colour, the abrupt visual change triggers a bottom-up attentional shift, reflexively drawing attention to the target location. Thus, SpeedPad performance likely depends on the interaction of these two processes: top-down preparation (maintaining readiness) and bottom-up orienting (reflexively shifting attention to the salient target). This combination enables participants to detect, orient to, and respond to targets efficiently, highlighting its relevance for real-world athletic tasks that require both anticipation and rapid reaction following attentional orienting to a stimulus.

Notably, our results also indicated that the ability to track moving stimuli, as measured with the MOT task, also predicted SpeedPad performance. While this result may initially appear counterintuitive, it could reflect the underlying role of split attention, which is a prerequisite for tracking multiple moving targets. Indeed, we believe that splitting attention across multiple locations likely contributes to efficient SpeedPad performance. The role of split attention, particularly at larger set sizes, mirrors the demands athletes face when visually tracking multiple moving players or objects during games (e.g., in soccer or basketball), where attentional capacity must adapt dynamically (4). This further highlights the ecological validity of SpeedPad as a training tool. The dominance of attentional orienting at smaller set sizes highlights the importance of quickly detecting and shifting attention to isolated targets. However, the predictive role of MOT accuracy in larger SpeedPad set sizes likely reflects the increasing demands for splitting attention across multiple target locations as array size grows. This interpretation is corroborated by the finding that MOT accuracy did not predict SpeedPad performance at set size 9, where the demands for splitting attention are minimal because all locations are clearly perceptible without shifting focus from the centre. At set size 9, the small number of discs reduces the need to split attention because all targets are easily visible without requiring multiple attentional shifts.

Contrary to our expectations, visual search performance did not significantly predict SpeedPad performance in any set size, despite the observed correlations. While visual search efficiency, indexed by the slope of the display size function, correlated with SpeedPad performance, it did not explain unique variance in the regression models. This likely reflects differences in task demands. Although SpeedPad requires one to search for the target in the array, that search is of a different kind than the one evaluated by the visual search task we used. In SpeedPad, the target disc lights up in a distinct colour and “pops out” of the array, creating a feature search scenario. Past research indicates that feature searches occur in parallel, allowing individuals to process all locations simultaneously and locate the target efficiently, regardless of distractor quantity. In contrast, the visual search task we used involved configuration searches, where the target differed from distractors on a spatial combination of features. Configuration searches are more demanding, requiring a two-stage process of initial parallel processing followed by serial processing (21). These searches typically exhibit a display size effect, where search time increases as the number of distractors grows, a result we clearly documented in the visual search data. While visual search efficiency correlated with SpeedPad performance, it did not uniquely predict it, likely because the general ability to process visual arrays efficiently plays a role in SpeedPad but does not map directly onto the task's reliance on feature-based pop-out detection. That said, it is worth noting that SpeedPad performance also decreased as the number of discs in the array increased. However, we propose that this decline reflects increased physical demands, such as larger arm movements, and the eye movements needed to visually scan larger arrays, rather than difficulty in shifting attention during search. Future studies could also explore this idea by introducing a conjunction search condition in SpeedPad, where additional distractor discs light up in different colours.

By identifying the specific attentional mechanisms underlying light training, our study provides a clearer understanding of how these tools may enhance reactive performance, addressing a gap in prior work that overlooked these cognitive processes. Overall, our findings provide novel insights into the cognitive processes underlying light training tasks, revealing a clear role for attentional orienting and split attention. Attentional orienting enables rapid detection and shifting of attention to targets, while split attention becomes increasingly important as task complexity grows. While previous studies (7–9) have demonstrated the discriminatory and performance-enhancing potential of light training tools, our findings clarify two specific cognitive processes they tap into, namely attentional orienting and split attention.

Beyond the strengths of this study, we note two potential limitations. First, given that the main goal was to investigate the underlying cognitive mechanisms of light training, we did not restrict our sample to participants doing sports. As a result, we ended up with a heterogeneous sample with varying experience with sports and physical exercise. Although we included engagement in physical activity as a dichotomous variable (i.e., engagement vs. no engagement) in statistical analyses, our design does not allow us to draw definitive conclusions about the role of sports engagement in light training performance. Although we have no reason to expect that the cognitive processes underlying light training would differ between athletes and non-athletes, future studies with more homogenous athlete groups (e.g., elite soccer players, amateur basketball players) could examine this possibility. Second, in our study we administered the 4 tasks to all participants in the same order, which could have elicited carryover of fatigue and/or practice effects. Nevertheless, the 3 computerized tasks were kept very short, involved minimal physical effort (i.e., pressing a key on the keyboard to indicate a response), and were separated by self-initiated breaks, thus we do not believe that these indeed caused fatigue that may have compromised the results. Being a task that could induce physical fatigue, SpeedPad was administered last to avoid carryover fatigue to the other tasks. Furthermore, given that the 3 computerized tasks tap on distinct cognitive processes, we consider it unlikely that there could be substantial carryover benefits or costs to performance from a preceding to a subsequent task. Even if that was the case, our statistical analyses on the data from each task yielded the expected patterns of results based on the literature, e.g., a cue validity effect in the Posner Cueing task, a display size effect in the Visual Search task, and an effect of set size in the MOT task. Nonetheless, we cannot safely rule out the possibility that the order effects were present, affecting overall performance in the 4 tasks and as a result the weights in the regression models.

Despite these limitations, our results document that attentional orienting and split attention underlie the execution of light training tasks, enhancing our understanding of how these tasks implicate mental skills. This deeper understanding highlights why such tools may improve reactive performance in sports contexts that require anticipation, attentional control, and rapid responses. These insights allow us to predict which cognitive measures are most likely to demonstrate improvements in future training studies and identify the types of sports activities that could benefit most from light training [see (22), for a discussion on cognitive training specificity in sports and Kalén, Bisagno, Musculus, et al. (23), for a relevant meta-analysis]. Future research could explore how targeted LED light training improves specific attentional processes, such as orienting and split attention, and whether these improvements translate into better performance in real-world sports tasks. The present study provides a first step into understanding how popular light training tasks such as the Batak Pro, the Fitlight Trainer, and SpeedPad implicate mental skills, allowing professionals in sports to use them in a more targeted manner, i.e., in athletes whose tasks in their sport entails splitting attention across various location and stimuli and orienting the focus of attention quickly towards abrupt stimuli. A logical next step is to investigate whether training with these tasks can improve the efficiency with which attention is split and directed, producing in turn, real-world benefits in performance.

## Data Availability

The raw data used for the analyses reported in this article are freely available at: https://osf.io/tp7s8.

## Footnotes

^1^It is not clear whether a Batak exercise could qualify as training agility as according to most definitions of agility, this requires whole-body change of direction in addition to rapid movement and change in the direction of the limbs [see (16) for a discussion].

^2^Results remain unchanged if we enter the RT for either valid or invalid trials.
